# Monodispersity of recombinant Cre recombinase correlates with its effectiveness *in vivo*

**DOI:** 10.1186/1472-6750-9-80

**Published:** 2009-09-11

**Authors:** Paola Capasso, Marisa Aliprandi, Giuseppe Ossolengo, Frank Edenhofer, Ario de Marco

**Affiliations:** 1 Cogentech - Consortium for Genomics Technologies, via Adamello 16, 20139 Milan, Italy; 2 Stem Cell Engineering Group, Institute of Reconstructive Neurobiology, University of Bonn, Life & Brain Center and Hertie Foundation Sigmund-Freud-Strasse 25, D-53105 Bonn, Germany

## Abstract

**Background:**

Cre recombinase is a common reagent used for the *in vivo *on/off switching of the expression of target genes flanked by *loxP *sites. In particular, recombinant TAT-Cre fusion constructs purified from bacteria have been used to promote the cell uptake of the enzyme. However, the recovery of active TAT-Cre remains a demanding process and its specific activity varies significantly among batches, making difficult data comparison.

**Results:**

We noticed a strong correlation between recombinase activity and enzyme monodispersity. The existence of such correlation enabled us to indirectly monitor the TAT-Cre recombinase activity during the multi-step purification process by measuring its monodispersity, a parameter detectable by means of a spectrofluorimetric assay that allows the calculation of the Aggregation Index (AI) in an easy and rapid way. AI values were recorded after each purification passage to identify the critical steps and to choose optimal alternatives for chromatographic conditions, desalting procedures, and protocols for bacterial endotoxin removal. Furthermore, the effect of metal ions and temperature on TAT-Cre aggregation and inactivation was characterized *in vitro*. Finally, we optimized the enzyme delivery protocol *in vivo *by following the accumulation tuning of the reporter protein β-catenin.

**Conclusion:**

A rational purification protocol for TAT-Cre has been developed by choosing the options that minimize the enzyme aggregation. Our data suggest that AI measurement should support the optimization of any protocol aiming at the recovery of monodispersed protein.

## Background

Cre recombinase from bacteriophage P1 is commonly used to induce specific recombination of DNA sequences between two *LoxP *recognition sites [1,2]. The technique is well established for *in vitro *applications [3] and it is increasingly used as a means to switch on/off genes *in vivo *to obtain conditional mutants in both cultured cells and model animals [4-6].

The *in vivo *effectiveness depends on two parameters: a) the preservation of the enzyme activity and b) its efficient uptake into the host cells. Cre recombinase is stable in the range 37-42°C and shows a low, but reproducible, permeability through cell membranes that can be strongly increased by the fusion to basic peptides like TAT, derived from HIV-TAT protein [6-8]. Other peptides have been proposed as fusion partners and were compared for their effectiveness in inducing Cre-dependent recombination *in vivo *[7,9]. Interestingly, all of them seem to reduce the structural stability of Cre recombinase [9].

So far, no manufacturer provides such reagents and the research groups use their self-produced material that tends to vary significantly in terms of both yields and recombinase activity among different purified batches. We investigated the relationship existing between enzyme activity and its structural stability to identify the chemiophysical factors responsible for the loss of Cre recombinase activity during the purification process. Their contribution to protein misfolding was detected by a simple and fast spectrofluorimetric method that evaluates protein aggregation [10]. Its reliability was validated by comparison with conventional analyses such as gel filtration and dynamic light scattering (DLS) and used to design an optimized purification protocol since TAT-Cre aggregation correlates with, and is predictive of, its recombinase activity.

## Results

### Purification parameters affecting TAT-Cre activity

The loss of enzymatic activity is usually correlated to the loss of native structure. Misfolded proteins tend to aggregate and can either precipitate or form soluble complexes. The identification of soluble aggregates is feasible by means of biophysical techniques. Among these, fluorimetric analysis [10,11] has the advantage of being fast and simple, and we already showed that it correlates with size exclusion chromatography [11]. In contrast to folded proteins, protein aggregates are characterized by elevated light scattering at 280 nm and low emission at 340 nm. The ratio between the two values (aggregation index, AI) can be used as an indicator of monodispersity, with high values indicating large aggregates and low values monodispersity.

**Figure 1:**
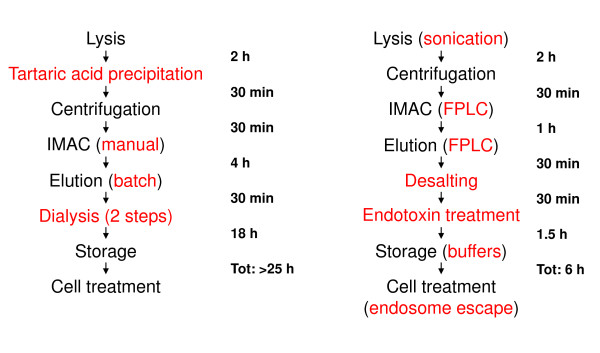
**Purification protocols**. Comparative flow-chart indicating steps and time requested in the original (left) and the alternative (right) protocol. The passages that have been tested for modifications are reported in red.

We purified a His-tagged TAT-Cre construct expressed in bacteria at either 4°C or 20°C according to the protocol published by Peitz et al. [12] (Figure 1). In both cases the protein obtained after affinity purification was almost homogeneously pure. A significant amount of the TAT-Cre purified at 20°C precipitated during dialysis in PBS and 10% glycerol. Insoluble aggregates were removed by centrifugation and the final yields of soluble TAT-Cre were 12 mg/L and 4 mg/L for purifications performed at 4°C and 20°C, respectively.

**Figure 2:**
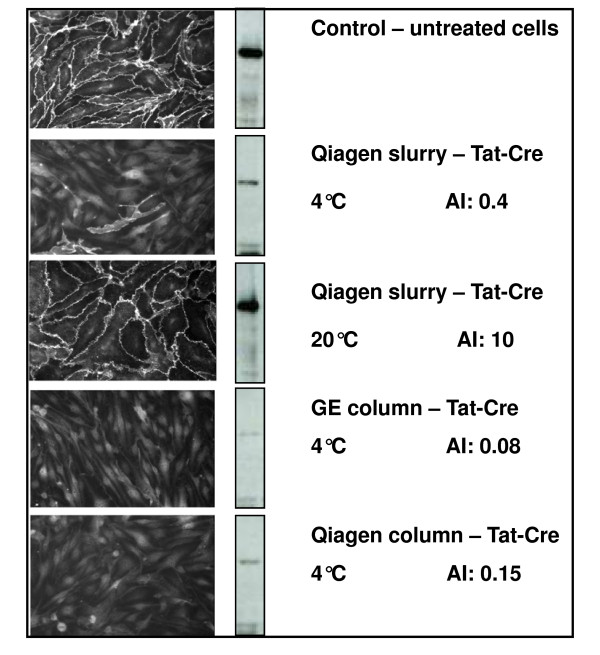
**In vivo recombinase activity of TAT-Cre with different level of soluble aggregation**. β-catenin was inserted between *loxP *sequences in endothelial cells and the effect of TAT-Cre recombinase addition (100 μg/mL) was analyzed 24 h after treatment by immunofluorescence on cultured cells and by WB evaluation of the β-catenin content. The untreated control was compared with cells incubated with enzyme samples purified using different protocols and scoring different AI values.

The recombinase activity was tested checking the decrease of β-catenin accumulation in endothelial cells. The protein accumulates in the extracellular matrix and its expression can be silenced by knocking out its DNA sequence inserted between two *LoxP *sites. The read out is easily revealed by immunofluorescence (IF) and quantified by WB analysis using cell lysates (Figure 2).

**Figure 3:**
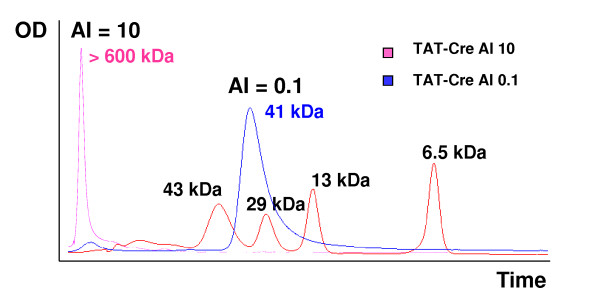
**TAT-Cre monodispersity evaluation by gel filtration and aggregation index (AI)**. Affinity purified TAT-Cre samples were assessed for their fluorimetric AI values and immediately processed by gel filtration.

Soluble TAT-Cre purified at 4°C had an AI value of 0.4, indicative of monodispersity, and was active. As shown in Figure 2, the amount of β-catenin in the cells, visualized by both IF and WB, was strongly reduced after TAT-Cre treatment. In contrast, when the same enzyme was purified at 20°C, its AI value was 10 and no significant recombinase activity was detectable (Figure 2). The separation of the two TAT-Cre samples by gel filtration confirmed that the enzyme purified at 4°C was a monomer, while the sample purified at 20°C formed large aggregates (Figure 3), according to the estimations of the fluorimetric assay. Therefore, the first conclusions were that TAT-Cre monodispersity is a suitable parameter to infer protein recombinase activity and that monodispersity evaluation by AI is a reliable method that can substitute the time consuming gel filtration chromatography.

Next we measured the Tm of the purified, active TAT-Cre. The relative high value (52°C) indicates that, although temperature control is a crucial factor to preserve TAT-Cre recombinase monodispersity and activity, the contribution of other process parameters may be crucial during the purification. We were also interested in simplifying the original purification protocol (Figure 1) and, consequently, decided to systematically measure the AI values at every single purification step to identify passages critical for preserving Cre recombinase activity.

First, the possibility of substituting the batch affinity chromatography with an FPLC-based alternative was evaluated. Both Qiagen and GE ready-made columns were tested and the results did not show significant differences in terms of AI values and recombinase activity when TAT-Cre was purified following the two protocols and using the different resins (Figure 2). However, the FPLC option allowed for an automated and faster (90 min instead of more than 4 h) purification. Furthermore, protein could be more extensively washed and eluted in a smaller volume and thus at a higher concentration.

The initial success prompted us to evaluate, with the help of the AI analysis, further protocol simplifications. However, EDTA (2 mM) addition in the buffer could not substitute the tartaric acid precipitation and the AI significantly increased when a desalting column was used for buffer exchange as an alternative to the time-consuming, two-step dialysis. Both modifications resulted in purified TAT-Cre with very low enzymatic activity (data not shown).

### Endotoxin removal

Even though it was not possible to find reliable alternatives to some time-consuming steps, the results confirmed the direct correlation between AI-evaluated monodispersity and recombinase activity. Therefore, AI analysis was used to optimize the endotoxin removal step and the composition of the storage buffers. Proteins purified from bacteria are commonly contaminated with endotoxins and such molecules are incompatible with several experimental designs. We compared three methods for endotoxin removal from samples of purified TAT-Cre, one based on two-phase partition [13], the second on ion-exchange chromatography, whilst in the third case a commercial affinity chromatography column has been used [14]. In the first instance, AI indicated a dramatic aggregation increase and the *in vivo *assay confirmed the loss of enzymatic activity. On the contrary, both chromatographic protocols prevented the protein aggregation, TAT-Cre remained fully active, and both the coagulation test and the induction of the endotoxin-marker genes MIP2 and IK-Ba in primary macrophages indicated a significant endotoxin concentration reduction in comparison to the untreated samples (data not shown).

The addition of 50% glycerol was sufficient to preserve the TAT-Cre activity for at least 6 months, although the addition of 50 mM trehalose was useful to stabilize enzyme batches that underwent more thawing/freezing cycles.

### *In vitro *effect of chemiophysical perturbations on TAT-Cre monodispersity and recombinase activity

Next we analyzed *in vitro *what chemio-physical factors were relevant for TAT-Cre stability. Protein aggregation was evaluated by both AI and DLS, an analytical technique routinely used for measuring protein monodispersity/polydispersity [15]. The estimation of the protein aggregation complexity performed by AI and DLS were always in good agreement (Tables 1 and 2).

**Table 1:** TAT-Cre recombinase *in vitro *stability in the presence of metal ions

	**DLS** **(R_H _in nm)**	**AI** **(280_em_/340_em _nm)**
Control	3.98 - monodispersed	0.02

1 mM NiCl_2 _at 4°C	polydispersed	8.5

10 mM NiCl_2 _at 4°C	polydispersed	9.2

1 mM NiCl_2 _at 20°C	-	precipitated

1 mM NiCl_2 _at 30°C	-	precipitated

0.1 mM NiCl_2 _at 4°C	polydispersed	3.1

0.01 mM NiCl_2 _at 4°C	4.15 - monodispersed	0.4

1 mM NiCl_2 _+ 5 mM EDTA	polydispersed	3.3

1 mM NiCl_2 _+ 10 mM EDTA	polydispersed	2.2

1 mM NiCl_2 _+ 200 mM TA	3.92 - monodispersed	0.02

Enzyme monodispersity was analyzed by dynamic light scattering (DLS) and fluorimetric aggregation index (AI). Purified recombinant protein samples (50 μg/mL) were incubated 30 min at different temperatures in the presence of increasing NiCl_2 _concentrations and of the chelators EDTA and tartaric acid (TA). DLS: the hydrodynamic radius (R_H_) of the molecules in solution is expressed in nm; the AI is the ratio between the light scattering at 280 nm and the emission at 340 nm. Experiments have been performed in triplicate.

**Table 2:** Effect of physical treatments on the stability of purified TAT-Cre recombinase

	**DLS** **(R_H _in nm)**	**AI** **(280_em_/340_em _nm)**
Control	3.98 - monodispersed	0.02

30' at 20°C	3.77 - monodispersed	0.04

60' at 20°C	3.87 - monodispersed	0.04

4 h at 20°C	3.94 - monodispersed	0.06

30' at 30°C	4.01 - monodispersed	0.06

10' at 40°C	3.96 - monodispersed	0.06

Sonication at 4°C	polydispersed	10

Sonication at 20°C	polydispersed	14

Sonication at 4°C + Trehalose	polydispersed	3.7

Sonication at 4°C + NiCl_2_	polydispersed	46.7

Sonication at 4°C + TA	polydispersed	2.5

Sonication at 4°C + NiCl_2 _+ TA	polydispersed	4.7

The effect of increasing temperature, sonication, trehalose (50 mM), NiCl_2 _(1 mM), and tartaric acid (200 mM) on the enzyme monodispersity was analyzed by dynamic light scattering (DLS) and fluorimetric aggregation index (AI) using purified recombinant protein samples (50 μg/mL). DLS: the hydrodynamic radius (R_H_) of the molecules in solution is expressed in nm; the AI is the ratio between the light scattering at 280 nm and the emission at 340 nm. Experiments have been performed in triplicate.

Metal ions may leak from resins used for affinity chromatography [16] and induce protein oxidation and consequent misfolding. For such reason we have compared the suitability of different commercially available columns (Figure 2) and the direct effect of NiCl_2 _on TAT-Cre monodispersity (Table 1). Traces of metal ions (0.1 mM of NiCl_2 _in the presence of 50 μg/mL of purified enzyme) were sufficient to induce the partial aggregation of the enzyme. EDTA was added to chelate free ions but, even at the highest concentrations compatible with affinity chromatography (5 mM), it was able to mitigate only partially the destabilizing effect of metal ions. In contrast, 200 mM solution of tartaric acid completely prevented the Ni ion-dependent TAT-Cre aggregation and was still compatible with affinity chromatography.

Temperature increase above 4°C resulted in TAT-Cre aggregation during purification and an incubation step of 10 min at 30°C was sufficient to induce its aggregation during two-phase partition in the presence of the detergent (data not shown). However, we found that the Tm of the purified enzyme was 52°C and that it was insensitive to temperature in the range of 20°C-40°C (Table 2). Incubation at 20°C for 4 h or at 40°C for 10 min did not modify the protein monodispersity, suggesting that co-factors might be necessary for the TAT-Cre's structural destabilization. Our data suggest that the presence of metal ions could catalyze a temperature-sensitive aggregation process that is inhibited by the addition of the tartaric acid (Table 1).

Also sonication seems to be a critical factor triggering the aggregation process, at both 4°C and 20°C (Table 2). Trehalose, a chemical additive used to stabilize protein structure [17], could only partially limit the sonication-dependent aggregation process. In contrast, the addition of metal ions induced a synergistic effect on the process of TAT-Cre aggregation, whilst the scavenging activity of tartaric acid limited the TAT-Cre sonication-induced aggregation.

### TAT-Cre cell-uptake and release from endosome

The *in vivo *enzymatic activity of Cre recombinase relies on the efficiency of two processes, namely its uptake into the cells and its release into the cytoplasm.

**Figure 4:**
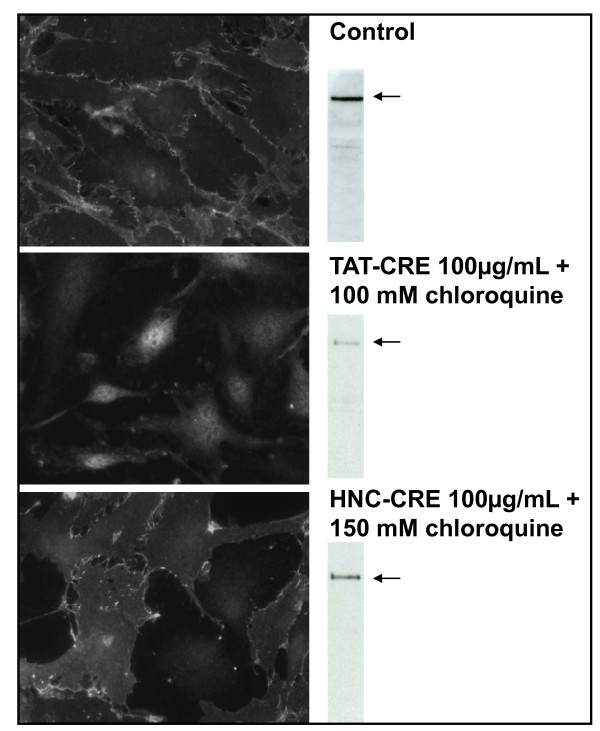
**Comparison of the recombinase activity of the constructs TAT-Cre and HNC**. β-catenin expression was knocked-out by the recombinase activity of either TAT-Cre or HNC (both 100 μg/mL) in the presence of chloroquine (0-100 μM).

Different constructs have been proposed for promoting the Cre recombinase uptake into the cells [9,12]. As an alternative to the TAT-dependent uptake, a construct in which Cre was fused to 6×His plus a nucleus leader signal (HNC) was designed and described as being even more efficient than TAT-Cre *in vivo *[9]. However, in our hands no active enzyme was recovered using the purification protocol described in the original paper. AI analyses indicated that HNC formed soluble aggregates. In contrast, when the same protocol optimized for purifying the TAT-Cre construct was used, 3-5 mg/L of monodispersed HNC recombinase were purified. Monodispersed HNC and TAT-Cre were compared for their recombinase activity *in vivo *by using the β-catenin assay, but the HNC construct resulted by far less active of TAT-Cre (Figure 4).

**Figure 5:**
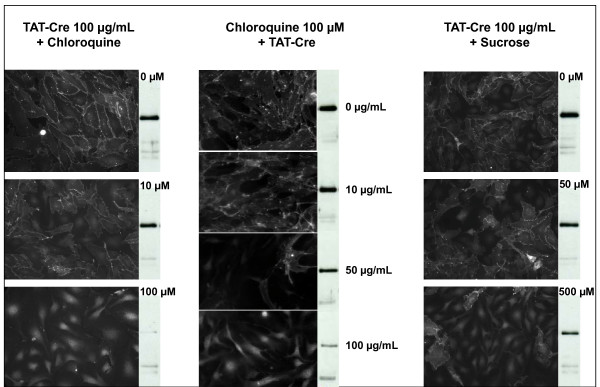
**Effect of chloroquine and sucrose addition on the TAT-Cre release from endosomes**. The β-catenin expression silencing induced by 100 μg/mL TAT-Cre in the presence of increasing concentrations of chloroquine (0-100 μM) and sucrose (0-500 μM) was evaluated by immunofluorescence. Similarly, the Cre recombinase activity of increasing TAT-Cre concentrations (0-100 μg/mL) was examined in the presence of 100 μM chloroquine.

**Figure 6:**
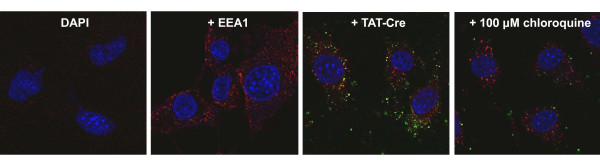
**TAT-Cre recombinase uptake: endosome transport and enzyme release**. Confocal microscopy was used to demonstrate the co-localization of GFP-labeled TAT-Cre recombinase (100 μg/mL) and the antibodies against the Early Endosome Antigen1 (EEA1). The concomitant addition of chloroquine (100 μM) resulted in the disappearance of Alexa488-containing endosomes. The cells were fixed 10 min after Cre treatment and the pictures correspond to DAPI only staining, DAPI + anti-EEA1, and the same plus Alexa488-TAT-Cre in the absence or in the presence of choroquine, respectively.

Polypeptides fused to TAT sequence remain trapped in the endosomes after cell-uptake by macropinocytosis [6,18] and several strategies have been proposed for inducing their leakage and protein accumulation into the cytoplasm. We confirmed that 100 mM chloroquine [18,19] was effective in making TAT-Cre recombinase available inside the cells (Figure 5). However, chloroquine cytotoxicity [18] prevents its use in sensitive cell lines. Therefore, sucrose [20] was used as an alternative, although it showed lower efficiency even in optimized conditions (Figure 5). Performing confocal microscopy using antibodies against the Early Endosome Antigen1 (EEA1, red signal) and Alexa488-labeled TAT-Cre (green signal), it was possible to confirm [18] the rapid uptake (less than 10 min) of the enzyme trapped into the endosome vesicles (Figure 6, yellow signal), and a substantial disappearance of TAT-Cre-filled vesicles in the presence of chloroquine treatment (Figure 6).

## Discussion

The Cre recombinase-dependent inducible knock-out of specific genes sided by *LoxP *sites is a methodology of constantly increasing interest [21]. Although recently an increasing effort has been devoted to improve the activity control of the Cre recombinase [22-25], a simple and reliable methodology for optimizing the protein purification is always profitable due to the fact that a major technical drawback remains the unavailability of commercial enzyme produced under standard conditions and the consequent variable activity of the home-made batches.

We first discovered that the enzymatic activity loss correlated with protein aggregation and that the AI was a reliable analytical indicator for immediate estimation of the Cre monodispersity after each single purification step. The AI is simple and fast to determine, showed a good agreement with other conventional analytical methods for protein aggregation such as gel filtration and DLS and, what is more, gives a value that is useful to compare the efficiency of different TAT-Cre purification batches and their stability during storage. It became, therefore, straightforward to predict the recombinase enzymatic activity at any time without the need to wait for the morphological/physiological readout of the treated cells. Furthermore, it became also possible to verify what purification steps and conservation conditions were deleterious for Cre native structure and, consequently, for its activity. Metal ions may be stripped out of the column [16] and we showed that they have the major responsibility for the enzyme aggregation. As a consequence, their inadequate removal resulted in the activity loss. Tartaric acid did not interfere with affinity purification even at concentrations of 200 mM and scavenged metal ions in an extremely more efficient way than EDTA, a conventional chelator used in buffers but tolerated at a maximal concentration of 5 mM in ion metal affinity chromatography. Furthermore, mild sonication conditions (see Methods) were adopted following the observation that such a treatment enhanced Cre aggregation.

Although the monodispersity of TAT-Cre purified from independent bacterial cultures could slightly vary, batches with AI values below 0.2 had high and comparable activity, namely the β-catenin content in treated cells became almost undetectable by WB 24 h after recombinase addition.

Once determined the optimal purification protocol and a reliable method for protein aggregation analysis, it became also feasible to compare the *in vivo *efficiency of different, but all monodispersed, Cre recombinase constructs and to show that a combination of TAT-Cre and chloroquine treatment assured the best recombinase activity *in vivo*. Preliminary results indicate that monodispersed TAT-Cre is suitable for *in vitro *recombination as well (data not shown).

## Conclusion

In order to be effective for *in vivo *treatment, Cre recombinase must be enzymatically active and efficiently translocated into the nucleus. We first found out that aggregation prevented the protein activity and we identified the factors leading to protein aggregation followed by the optimization of the protocols for TAT-Cre purification and cell treatment. We expect that the described methodology based on the use of the AI will be generally suitable for setting purification protocols and improving their quality evaluation and reproducibility. For its simplicity, it should be particularly convenient for labs not equipped with specialized biophysical instrumentation.

## Methods

### Cre recombinase purification

TAT-Cre was purified by modifying the original method described by Peitz et al. [7,12]. Bacterial pellets were resuspended in lysis buffer (50 mM Na_2_HPO_4_, 5 mM Tris pH 7.8, 500 mM NaCl, 1 mM PMSF) and incubated with lysozyme 1 mg/mL (Sigma) and DNase 100 μg/mL (Roche). The samples were sonicated using a water bath Bioruptor from Diagenode (30 s on/30 s off pulses, at low intensity for 15 minutes) and tartaric acid was added at a final concentration of 1 M. After incubation (5 min), the samples were centrifuged 45 min at 40000 rpm and filtered using a 0.22 μm filter (Millipore) before their loading on a 5 mL His-Trap HP column (GE Healthcare) or a Ni-NTA column (Quiagen) equilibrated in 50 mM Na_2_HPO_4_, 5 mM Tris pH 7.8, 500 mM NaCl.

Proteins were eluted in 50 mM Na_2_HPO_4_, 5 mM Tris pH 7.8, 500 mM NaCl, 300 mM imidazole, dialyzed in 20 mM HEPES, pH 7.4, 50% glycerol, 500 mM NaCl, and final samples were sterilized by filtration using Millex-GV filters (Millipore).

Cre-Rec (HCN) was cultured according to the conditions described by Lin et al. [9] and purified using the same protocol developed for TAT-Cre.

Trehalose (50 mM) was tested as an alternative to glycerol for storing of the purified Cre recombinase resuspended in 20 mM HEPES pH7.4 and 600 mM NaCl.

### Cell culture and Cre recombinase activity *in vivo *and *in vitro*

#### Cell Culture

β-catenin flox-endothelial cells were grown at 37°C in DME, 20% FCS, 100 μg/mL heparin, 5 μg/mL EC growth supplement (homemade from calf brain), 100 U/L penicillin/streptomycin, 2 mM glutamine, and 1 mM Na-pyruvate in the presence of 5% CO_2_.

#### Cre recombinase activity

Endothelial cells cultured in serum-free DME were treated with either TAT-Cre or HNC (0-150 μg/mL) for 1 hour and then incubated 30 min in the presence of chloroquine (0-100 μM) or sucrose (0-500 mM). Following a wash in DME, cells were cultured in complete medium for 24 hours.

#### Immunofluorescence

80,000 cell/well were seeded on coverslips coated with 0.5 mL of gelatin solution using 24-well plates. The day after slides were rinsed three times in PBS, fixed with 4% formaldehyde, and permeabilized by 5 min incubation in PBS plus 0.5% Triton X-100. Cells were successively washed three times, blocked with 2% BSA, and incubated in the presence of anti-β-catenin monoclonal antibody diluted 1:100 in PBS/2% BSA. Donkey-anti mouse-cy3 was used as a secondary antibody. After DAPI incubation (3 min) the slides were mounted and cells assessed by fluorescence microscopy at the DAPI and cy3 channels.

#### Confocal microscopy

Cells seeded on coverslips were first incubated with TAT-Cre labeled with Alexa488 in serum-free DME and then in the presence of 100 μM chloroquine for 10-60 min. Slides were rinsed, fixed and permeabilized with 0.1% saponin in PBS before being incubated 60 min in the presence of anti-EEA1 (Santa Cruz Biotechnol) diluted 1:150 in PBS containing 2% BSA and 0,05% saponin. Donkey-anti mouse-cy3 was used as a secondary antibody. After DAPI incubation (3 min) the slides were mounted and images were acquired at 405-,488-, 633-nm laser lines using a confocal microscope (SP2, Leica).

#### Immunoblot analysis

Confluent cells were washed with ice-cold PBS and scraped in lysis buffer. Samples were separated by SDS PAGE (12%), transferred to nitrocellulose membranes (0.45 μM, Whatmann), and probed with anti-β-catenin monoclonal antibodies (BD Biosciences) diluted 1:500 in PBS containing 5% dry milk. The secondary antibody was an anti-mouse HRP-conjugated (BIO-RAD) diluted 1:20000 in PBST. Detection was carried out by chemioluminescence (ECL, GE Healthcare).

### Evaluation of protein aggregation

#### Size Exclusion Chromatography (SEC)

The apparent mass of TAT-Cre batches showing different level of aggregation was evaluated at 4°C using HiLoad16/60 Superdex 200 and Superose 12 10/300 GL columns coupled to ÄKTA Explorer (GE Healthcare). The running buffer was 20 mM HEPES, pH 7.4, 600 mM NaCl, and the protein molecular mass was estimated using a low molecular weight calibration kit (GE Healthcare).

#### Dynamic Light Scattering (DLS)

The measurements were performed at 18°C by employing a DynaPro 99 (Protein Solutions) instrument and using the Dynamics 5.20.05 software for data evaluation (Protein Solutions).

Aggregation Index (AI). The aggregation index [10] of Cre recombinase samples was evaluated after having recorded the signal between 260 and 400 nm and calculated the ratio between the value of scattered light at 280 nm and the emission peak at 340 nm. The fluorescence measurements were performed using an AB2 Luminescence Spectrometer (Aminco Bowman Series 2) equipped with SLM 4 software and a J-810 spectrofluorimeter (Jasco).

#### Determination of the protein melting temperature (Tm)

First far UV circular dichroism spectra of folded proteins were acquired using a J-810 spectrofluorimeter (Jasco). The wavelength corresponding to the maximal signal variation with respect to the zero was chosen and the value of molar ellipticity was followed while the temperature was constantly increased from 20°C to 100°C. Tm was calculated as the intersection between the midpoint transition line and the ellipticity curve obtained as a function of the temperature.

### *In vitro *TAT-Cre stability tests

Purified recombinant protein samples (50 μg/mL in 20 mM HEPES, 150 mM NaCl, 5% glycerol) were used at different temperatures and incubation times, as specifically indicated in the Table legends. NiCl_2 _was used at concentrations between 0.01 and 10 mM, EDTA at 5-10 mM, tartaric acid at 200 mM, and trehalose at 50 mM. Samples were sonicated at 4°C in a water bath (5 times × 20 sec at medium intensity) using a Diagenode Bioruptor.

### Endotoxin removal

Ion chromatography. Purified TAT-Cre was loaded onto a MonoQ 4.6/100 PE (GE Healthcare) and eluted with a linear gradient 150 mM - 1 M NaCl. Fractions containing TAT-Cre were pooled and checked for endotoxin content (see below).

Affinity chromatography. Purified TAT-Cre was loaded onto an Endotrap Blue 1/1 column (Profos) and recovered according to the manufacturer's instructions.

Two-phase partition. Endotoxin removal from TAT-Cre preparation by using two-phase partition in the presence of Triton X-114 was performed following the protocol described by Liu et al. [13].

The endotoxin concentration was estimated using the LAL-Gel Clot Pyrogent Plus Kit from Cambrex. The *in vivo *evaluation of effective endotoxin removal was performed by quantifying the expression of the stress markers MIP2 and IK-Ba in primary macrophages.

## Competing interests

The authors declare that they have no competing interests.

## Authors' contributions

PC set the experimental conditions and performed purifications and endotoxin experiments, MA investigated the *in vivo *enzymatic activity, GO optimized the chromatographic protocols, FE provided the starting material and protocols and contributed to manuscript writing, AdM performed the *in vitro *experiments and drafted the final manuscript. All authors approved the final manuscript.
